# Impact of Water Management on Growth and Pigment Composition of Cauliflower and Broccoli

**DOI:** 10.3390/plants14050725

**Published:** 2025-02-27

**Authors:** Fatemeh Izadpanah, Navid Abbasi, Forouzande Soltani, Susanne Baldermann

**Affiliations:** 1 Leibniz-Institute of Vegetable and Ornamental Crops, Food Chemistry and Human Nutrition, Theodor-Echtermeyer-Weg 1, 14979 Großbeeren, Germany; izadpanah_f@yahoo.com; 2 Institute of Nutritional Sciences, Food Chemistry, University of Potsdam, Arthur-Scheunert-Allee 114-116, 14558 Nuthetal, Germany; 3 Department of Horticultural Science, University of Tehran, Daneshkade Ave, Karaj 31587-77871, Iran; navida.0890@gmail.com (N.A.); soltanyf@ut.ac.ir (F.S.); 4 Faculty of Life Sciences: Food, Nutrition and Health, Food Metabolome, University of Bayreuth, Fritz-Hornschuch-Straße 13, 95326 Kulmbach, Germany

**Keywords:** climate change, drought stress, irrigation, cauliflower, broccoli, *Brassica oleracea*, carotenoids, chlorophylls

## Abstract

Global climate change minimizes fresh water resources used in agriculture worldwide. It causes drought stress, which has adverse effects on plants. To ensure food security, crops and vegetables capable of tolerating shortages of water over the growth period are needed. This study aimed to elucidate the morphological and biochemical responses of three colored cauliflower (*Brassica oleracea* var. *botrytis*) cultivars (Clapton, Trevi, and Di Sicilia Violetto) and one broccoli cultivar (*Brassica oleracea* var. *italica* var. *Magic*) to different irrigation treatments (85–100%, 65–80%, 45–60%, and 25–40% field capacity). Assessment of growth parameters revealed no significant difference among all the treatments for root fresh weight, leaf area, and floret size. Major water shortages reduced the floret and stem fresh weight of the Clapton cultivar. Additionally, under severe drought stress, only the Di Sicilia Violetto cultivar had a decrease in plant height, but no impact on the number of leaves was observed. The measurement of pigment contents in the leaves showed no significant difference in carotenoids in all the cultivars; just the chlorophyll contents decreased with moderate stress in the Di Sicilia Violetto cultivar. This research demonstrates that cauliflower and broccoli are likely drought-tolerant vegetables and common irrigation regimes may be reviewed.

## 1. Introduction

Global climate change affects rainfall patterns [1], causing plants to suffer from drought in many parts of the world, especially in arid and semi-arid regions [2]. Water scarcity, followed by drought stress, poses serious challenges to agricultural productivity and future food security [3,4,5]. According to reports, the agricultural sector consumes roughly 70% of global freshwater consumption [6], but water use efficiency in many countries is less than 50% [6,7]. Drought stress is one of the detrimental consequences of climate change, negatively affecting crops’ morphological, physiological, and biochemical factors and resulting in crop productivity reductions [8]. Hence, finding solutions to reduce water use and selecting drought-tolerant crops and vegetables are major issues in sustainable agriculture [9]. Addressing these issues requires more effective methods: firstly, it is important to improve irrigation management by means of optimal irrigation strategies and water-efficient growing methods or controlled environment cultivation as an alternative to traditional field-based farming (surface irrigation) [5,10]. Secondly, there is a need to find drought-tolerant plant species without yield loss. To cope with drought conditions, employing a combination of strategies to improve plant drought resistance might be necessary [11].

Over the past two decades, the global agricultural value surged by 89 percent, while agriculture’s contribution to the global economic output remained relatively stable in 2022 (FAO 2024) [12]. With a global production of 26.5 million tons and 59,776.8 tons in Iran (FAO, 2023) [13], cauliflower (*Brassica oleracea* var. *botrytis*) and broccoli (*Brassica oleracea* var. *italica*) are among the most important and consumed vegetables of the Brassicaceae family over the world and specially in Iran [14,15,16]. Both are good sources of bioactive phytochemicals, like carotenoids, glucosinolates, fibers, and other macro- and micronutrients [17,18]. Thus, they have human health benefits including anti-cancerous properties, as well as preventing and treating cardiovascular diseases and eye-related disorders [17,19,20,21].

Cauliflower and broccoli grown in an open field and with traditional irrigation generally use large amounts of water, and water scarcity would be one of the main problems in the growth and production of these crops if this conventional farming practice is to be maintained [22,23]. Many studies report the effect of drought stress on physiology and secondary metabolite contents in different plant species and also in *Brassica oleracea* L. [24,25,26,27,28,29,30,31,32]. For instance, a case study evaluating the impact of drought revealed that the area for Brassica vegetables has decreased by 3.2% on an annual basis. The highest negative standardized yield of Brassica vegetables in relation to all vegetables in the study, as a result of severe drought, has been recorded in the Czech Republic over the past decades [33]. In this context, the main objective of this study was to find out the impact of water shortages on different colored (white, green, and purple) cauliflower and broccoli in a controlled cultivation trial.

## 2. Materials and Methods

### 2.1. Plant Growth Conditions and Drought Stress Application

The experiment was carried out in 2020–2021 at the experimental station of Tehran University (latitude 35°48′, longitude 50°57′, and 1320 m above sea level), Iran. Three colored cauliflower cultivars (white: Clapton, green: Trevi, and purple: Di Sicilia Violetto), and one broccoli cultivar (Magic) were used for the experiment. The seeds of Trevi (green), Di Sicilia Violetto (purple), and Magic (broccoli) cultivars were purchased from Hazera Seeds GmbH Company (Edemissen, Germany), and ‘Clapton’ (white) from the Volmary GmbH Company (Münster, Germany). They were sown in sowing bowls containing standard soil (coco peat and perlite at a ratio of 1:1), and, after five weeks, transferred in the greenhouse. During the plants’ growth, temperature in the greenhouse ranged between 25 °C (minimum) and 37 °C (maximum), with an average of 31 °C. The average air relative humidity was 60%. The experiment was conducted in a randomized block design with three replicates and three plants in each replicate. Soil samples were collected from 0–30 cm depths and analyzed in the Soil Science Laboratory of the Department of Irrigation and Reclamation Engineering at the University of Tehran, Iran. Initial soil properties, including soil texture, available water content, field capacity (FC), permanent wilting point (PWP), and soil electrical conductivity (EC), were determined. Field capacity (FC) is the amount of soil moisture or water content held in soil after excess water has drained away [34]. The retrieved values were pH = 8, FC = 27.04%, PWP = 12.44%, and EC = 1.4 dS/m. The soil texture was classified as loamy with a composition of 22.54% clay, 31.66% silt, and 45.80% sand. The Penman–Monteith method, stage-specific crop coefficients, and meteorological data from a nearby weather station were used to estimate the ET_c_ values, according to formula (1) [35] as follows:

Et_c_ = ET_o_ × K_c_ (1)

where Et_c_ is the crop water requirements (mm), ET_o_ is the reference crop evapotranspiration, and K_c_ is the crop coefficient.

A Teta probe (TDR 100 Soil Moisture Meter, Spectrum Technologies, Inc., Plainfield, IL, USA) was used every 2 days to monitor the soil moisture in the treatments and measure soil moisture volume. To evaluate the drought stress, one week after transferring the transplants to the greenhouse (late November), four levels of irrigation, including T1—no stress as control, in which plants were watered well (85–100%) through daily irrigation; T2—low drought stress, which corresponded to 65–80% field capacity (FC); T3—moderate stress, which corresponded to 45–60% FC; and T4—severe stress, which corresponded to 25–40% FC, were applied, till the harvest time (beginning of February).

### 2.2. Plant Growth Parameters, Carotenoids, and Chlorophyll Measurements

At the end of the growth period, the whole plants were harvested and different growth parameters, such as floret fresh weight and size, fresh weight of stem and total roots, leaf number per plant, and plant height from floret to crown area, were recoded. To measure the leaf area on average, three full expanded leaves from each plant were separated, then measured by a leaf area meter (Model: DELTA-T DEVICES, Cambridge, UK) and the data recorded in square centimeters.

Carotenoids and chlorophylls were extracted as previously described by Frede and Baldermann (2022) [36]. Briefly, 5 mg of freeze-dried, powdered leaves for all cultivars were used for extraction with tetrahydrofuran/methanol (1:1, *v*/*v*) solvents and analyzed by a UHPLC-DAD-ToF-MS device (Agilent Technologies, Waldbronn, Germany). Identification was performed by comparison of the spectra and retention times with authentic standards and available reference data in the literature [37]. Quantification was done at 450 nm. The results were expressed in ng mg^−1^ DW (dry weight) as means ± standard error of three biological replicates from each cultivar.

### 2.3. Data Analysis

The collected data were analyzed using IBM SPSS Statistics for Windows Version 26.0 (IBM Deutschland, Ehningen, Germany). One-way ANOVA and Tukey HSD post hoc test were used to compare the means of different treatment groups for each cultivar separately. The normal distribution of data in the different samples was tested (Shapiro–Wilk). A *p*-value of ≤ 0.05 (95% confidence level) was considered statistically significant and results are presented as the means ± SE in graphs. Microsoft Excel 2019 was utilized to create graphs.

## 3. Results

### 3.1. Effect of Drought Stress on Growth Parameters

**Figure 1:**
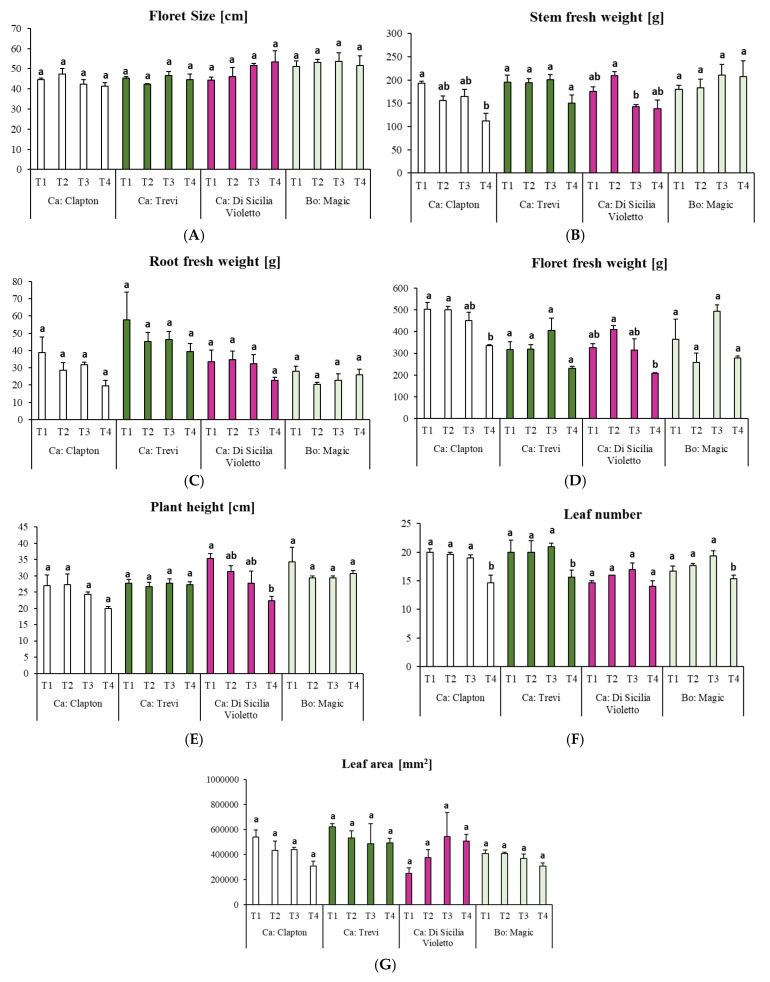
Effect of water regime (T1: watered well; T2: low stress; T3: moderate stress; and T4: severe stress) on (**A**): floret size; (**B**): stem fresh weight; (**C**): root fresh weight; (**D**): floret fresh weight; (**E**): plant height; (**F**): leaf number; and (**G**): leaf area parameters in *Brassica oleracea* var. *botrytis* and *Brassica oleracea* var. *italica* var. *Magic.* Each bar indicates means and standard error of three replicates by using one-way ANOVA at *p* < 0.05. Letters indicate statistically significant differences between different cultivars in alphabetical order from highest to lowest. Ca: cauliflower and Bo: broccoli.

In the present study, different morphological parameters such as floret size, fresh root weight, and leaf area showed no significant decrease or increase under different treatments and in all cultivars of cauliflower and broccoli (Figure 1A,C,G). Both floret and stem fresh weight parameters were significantly reduced compared to the control treatment by severe water deficit (25–40% FC) in the Clapton cultivar (Figure 1B,D). Although the severe drought stress decreased the plant height in the Di Sicilia Violetto cultivar, the number of leaves did not change in this cultivar (Figure 1E,F).

### 3.2. Effect of Drought Stress on Carotenoids and Chlorophylls

**Figure 2:**
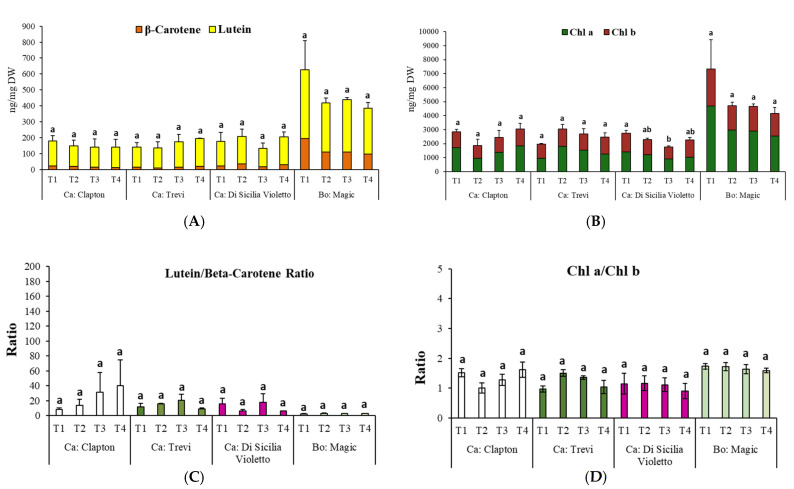
Effect of water regime (T1: watered well; T2: low stress; T3: moderate stress; and T4: severe stress) on (**A**): chlorophylls; (**B**): carotenoids accumulation; and (**C**): lutein/β-carotene. (**D**): chl a/chl b ratios in *Brassica oleracea* var. *botrytis* and Brassica oleracea var. *italica* var. *Magic*. Each bar indicates means and standard error of three replicates by using one-way ANOVA at *p* < 0.05. Letters indicate statistically significant differences between different cultivars in alphabetical order from highest to lowest. Ca: cauliflower and Bo: broccoli.

The HPLC analysis of the leaf extracts revealed the presence of two major carotenoids, β-carotene and lutein, in cauliflower and broccoli. Chlorophyll a and chlorophyll b were the two main chlorophylls investigated in this study (Figure 2A,B and Table S1). Under deficit irrigation levels, no significant effect on the content of β-carotene, lutein, chlorophyll a, and chlorophyll b was observed. Moderate drought stress significantly decreased the content of total chlorophylls, just in the purple cultivar Di Sicilia Violetto (from 2748.0 ± 343.2 to 1762.1 ± 126.1 ng mg^−1^ DW), and neither a positive nor a negative effect was observed in the other cultivars (Figure 2B and Table S1). Also, the contents of total carotenoids, lutein/β-carotene, and chlorophyll a/b ratio were not affected by different water regimes (Figure 2A,C,D).

## 4. Discussion

Water scarcity due to climate change and mismanagement in many parts of the world is a challenge to future food security and environmental sustainability [4]. Freshwater availability is expected to decrease dramatically as freshwater supplies become more scarce across the globe [38]. The FAO predicts that global water demand for agriculture will increase by 60% by 2050 to meet the growing food needs of a growing population [39,40]. In this regard, the development of water management practices such as optimizing irrigation scheduling and improving water use efficiency through the use of alternative water resources and efficient, localized irrigation systems will increase water use efficiency to sustain food production [4,41,42]. One of the most important abiotic stresses that severely affects the growth, yield, product quality, biosynthesis, and metabolism of secondary metabolites in *Brassica* crops is drought [43].

### 4.1. Drought Stress Effects on Physiological Parameters

Under water-limited conditions, a significant reduction in plant growth and yield production have been observed in different crops, such as canola [44], cauliflower [45], maize [46], and mungbean [47]. The discrepancy in the findings of the present study in comparison to that of cauliflower may be attributed to the variations in the experimental design. In the study of Latif, Akram, and Ashraf [45], seeds were treated with ascorbic acid, while in the present study, drought stress was induced by employing distinct water regimes.

It is expected that drought stress reduces growth parameters like leaf size, stem extension, and root proliferation in plants [48]. However, in this study, no significant effect was observed under mild and moderate drought stress in all the cultivars studied. A deeper understanding of growth in response to water limitation and the trade-off between conserving metabolic resources and ensuring adequate water access and uptake should be part of further studies, including adaptations at the cellular level and organ level or metabolic changes [49] using state-of the-art microscopic and instrumental–analytical methodologies such as single cell metabolomics [50]. Only under severe stress conditions was a reduction in yield observed for two cultivars, Clapton and Di Sicilia Violetto. The changes in our study are comparable with those reported by Kartika et al. [51].

The findings of this study demonstrate that the susceptibility of cauliflower and broccoli to water shortage is cultivar-specific. However, the study also indicates that targeted selection of cultivars, in combination with optimized water utilization strategies, has the potential to enhance the environmental sustainability of cauliflower and broccoli cultivation without significant yield reductions.

### 4.2. Drought Stress Effects on Photosynthetic Pigments

In plants, carotenoid pigments play a pivotal role in photosynthesis and provide the precursors for the biosynthesis of plant hormones like ABA and strigolactones, which are of great importance in plant stress tolerance [52,53]. Drought stress has been reported to affect the content of carotenoids in many crops, including strawberry [54], maize [55], Chinese flowering cabbage (Choysum), Chinese kale (Kailaan) [56], and soybean [57]. In the specific experimental conditions applied in this study, no alterations were observed in the β-carotene and lutein contents.

Furthermore, photosynthetic pigments (chlorophyll a and b) are essential for plant growth and development. Under drought stress, chlorophyll concentrations decrease through the synthesis of reactive oxygen species like O_2_ and H_2_O_2_ in plants [57,58]. It is reported that the accumulation of osmolytes such as proline in stress conditions could be a tolerance indicator in some plant species [46,59]. In this study, the total chlorophyll content in the cauliflower cultivars Clapton (white) and Trevi (green) and the broccoli cultivar (Magic) was not affected by water shortage or drought stress. One potential explanation for this phenomenon is that, due to the photosystem II’s (PSII’s) high tolerance to drought stress, it only exhibits a reaction under conditions that are particularly extreme, which were not yet observed in this study [60]. In addition, it has been documented that cauliflower exhibits a response to conditions of drought by decreasing photosynthesis, a process that arises from reduced gas exchange through stomatal conductance. This response, however, does not result in a decline in chlorophyll content [61]. However, other cultivars are more sensitive to drought. When subjected to moderate levels of stress, a slight decrease in chlorophyll content was observed in Di Sicilia Violetto. This outcome aligns with the documented impact of moderate deficit irrigation on leaf chlorophyll content in cabbage (*Brassica oleracea* L.) [62].

The present results suggest that growth is only marginally affected and that photosynthesis is only slightly altered, thus confirming the possible tolerance to water shortages in the studied cultivars of cauliflower and broccoli.

## 5. Conclusions

In conclusion, the present research demonstrates that the studied cultivars of cauliflower and broccoli are likely to be water shortage-tolerant vegetables. This finding is significant as it suggests that these crops can be cultivated in regions with limited water availability without substantial yield losses. Future research should concentrate on the optimization of irrigation practices during vegetable cultivation to maximize water use efficiency and crop productivity. The implementation of water-saving measures, such as drip irrigation and precise irrigation scheduling, is critical to enhancing food security, a challenge that remains at the global level. Furthermore, the development of more drought-resistant cultivars through breeding programs or genetic enhancements could significantly enhance the resilience of these crops to water stress across various experimental and environmental conditions in different regions. Exploring these potential future research avenues will contribute to advancing our understanding and implementation of effective strategies to mitigate the challenges posed by water scarcity.

## Data Availability

Data are contained within the article and Supplementary Materials.

## Supplementary Materials

The following supporting information can be downloaded at https://www.mdpi.com/article/10.3390/plants14050725/s1. Table S1: Carotenoid and chlorophyll content of the different cauliflower (Ca) cultivars and the broccoli (Bo) cultivar under different water regimes: T1: well-watered, FC 85–100%, T2: low stress FC 65–80%; T3: moderate stress FC 45–60%; and T4: severe stress FC 25–40%.
