# Supplementary material for: Impact of Water Management on Growth and Pigment Composition of Cauliflower and Broccoli
Source: Plants (Basel). 2025 Feb 27;14(5):725. doi: 10.3390/plants14050725 (PMC11901868; doi:10.3390/plants14050725)
Supplement: Supplementary file 1 [file plants-14-00725-s001.zip › plants-3463416-supplementary.pdf]

Impact of Water Management on Growth and Pigment Composition of Cauliflower and Broccoli

Fatemeh Izadpanah, Navid Abbasi, Forouzande Soltani and Susanne Baldermann

Tabel S1:

Carotenoid and chlorophyll content of the different cauliflower (Ca) cultivars and the broccoli (Bo) cultivar under different water regimes: T1: well-watered, FC 85-100%, T2: low stress FC 65-80%; T3: moderate stress FC 45-60%; and T4: severe stress FC 25-40 %.

Cultivar	Treatment	Carotenoids (ng/mg DW)			Chlorophylls (ng/mg DW)		
		β-Carotene	Lutein	Total	Chl a	Chl b	Chl a + b
Ca: Clapton	Control	22.3 ± 8.3 a	157.8 ± 25.1 a	179.9 ± 33.6 a	1705.8 ± 73.1 a	1148.1 ± 662.9 a	2583.9 ± 158.4 a
	65-80%	18.6 ± 9.9 a	130.4 ± 24.7 a	149.1 ± 34.0 a	953.7 ± 310.0 a	911.4 ± 526.2 a	1865.2 ± 443.1 a
	45-60%	14.9 ± 14.3 a	125.0 ± 38.3 a	139.9 ± 52.7 a	1386.5 ± 364.1 a	1058.7 ± 611.2 a	2445.2 ± 490.2 a
	25-40%	12.1 ± 11.7 a	128.6 ± 36.7 a	140.8 ± 48.4 a	1839.6 ± 180.3 a	1212.1 ± 699.8 a	3051.1 ± 405.1 a
Ca: Trevi	Control	15.2 ± 5.7 a	124.6 ± 21.8 a	139.8 ± 27.5 a	954.4 ± 26.3 a	1008.3 ± 582.1 a	1962.8 ± 59.7 a
	65-80%	8.1 ± 2.3 a	127.9 ± 35.6 a	135.7 ± 37.8 a	1825.1 ± 236.1 a	1211.2 ± 699.3 a	3036.3 ± 334.9 a
	45-60%	15.1 ± 9.3 a	157.4 ± 40.1 a	172.6 ± 47.9 a	1544.8 ± 184.5 a	1155.4 ± 667.1 a	2700.3 ± 361.1 a
	25-40%	20.4 ± 2.7 a	173.5 ± 4.7 a	194 ± 2.9 a	1253.0 ± 288.9 a	1204.1 ± 695.1 a	2457.1 ± 317.5 a
Ca: Di Sicilia Violetto	Control	20.8 ± 13.2 a	155.6 ± 45.2 a	176.5 ± 57.5 a	1417.0 ± 384.0 a	1330.9 ± 156.5 a	2748.0 ± 198.1 a
	65-80%	35.9 ± 17.8 a	171.2 ± 24.7 a	207.1 ± 47.5 a	1202.9 ± 121.0 a	1094.5 ± 137.3 a	2297.5 ± 89.1 ab
	45-60%	15.7 ± 7.5 a	117.8 ± 24.7 a	133.5 ± 32.3 a	903.4 ± 110.9 a	858.7 ± 105.9 a	1762.1 ± 72.8 b
	25-40%	29.4 ± 4.5 a	176.5 ± 31.2 a	205.9 ± 28.7 a	1030.9 ± 237.6 a	1229.2 ± 167.0 a	2260.0 ± 174.1 ab
Bo: Magic	Control	193.7 ± 64.9 a	431.8 ± 119.0 a	625.6 ± 183.7 a	4685.9 ± 1424.2 a	2639.7 ± 690.1 a	7325.7 ± 2113.4 a
	65-80%	109.1 ± 18.9 a	308.9 ± 13.3 a	418.1 ± 30.4 a	2978.2 ± 221.1 a	1733.9 ± 65.8 a	4712.2 ± 247.3 a
	45-60%	110.0 ± 1.18 a	327.9 ± 14.3 a	437.9 ± 13.3 a	2890.2 ± 195.3 a	1783. ± 79.4 a	4673.8 ± 159.7 a
	25-40%	95.8 ± 10.1 a	289.0 ± 26.9 a	384.8 ± 37.1 a	2551.0 ± 301.3 a	1600.9 ± 137.2 a	4151.9 ± 431.9a
